# *COL5A1* RS12722 Is Associated with Temporomandibular Joint Anterior Disc Displacement without Reduction in Polish Caucasians

**DOI:** 10.3390/cells10092423

**Published:** 2021-09-14

**Authors:** Bartosz Dalewski, Katarzyna Białkowska, Łukasz Pałka, Anna Jakubowska, Paweł Kiczmer, Ewa Sobolewska

**Affiliations:** 1 Department of Dental Prosthetics, Pomeranian Medical University, 70-111 Szczecin, Poland; bartosz.dalewski@pum.edu.pl (B.D.); rpsobolewski@wp.pl (E.S.); 2 Department of Genetics and Pathology, Pomeranian Medical University, 70-111 Szczecin, Poland; katarzyna.kaczm@gmail.com (K.B.); aniaj@pum.edu.pl (A.J.); 3 Private Dental Practice, 68-200 Zary, Poland; 4 Laboratory of Molecular Biology and Genetic Diagnostics, Pomeranian Medical University, 70-111 Szczecin, Poland; 5 Department and Chair of Pathomorphology, Faculty of Medical Sciences in Zabrze, Medical University of Silesia, 40-055 Katowice, Poland; pawel.kiczmer@protonmail.com

**Keywords:** TMJ, TMD, temporomandibular joint disc, type V collagen, *COL5A1*, rs12722, rs13946, TMJ disc displacement

## Abstract

Numerous reports describe the association between the single-nucleotide polymorphism (SNP) rs12722 and rs13946 in the *COL5A1* gene and injuries, such as Achilles tendon pathology, anterior cruciate ligament (ACL) injuries, and tennis elbow. Hence, there were no studies investigating *COL5A1* and temporomandibular joint (TMJ) pathology. The aim of this study is to evaluate the relationship between *COL5A1* rs12722 and rs13946 SNPs and TMJ articular disc displacement without reduction (ADDwoR). In this case-control study, the study group consisted of 124 Caucasian patients of both sexes. Each patient had a history of ADDwoR no more than 3 months prior. The control group comprised 126 patients with no signs of TMD according to DC/TMD. Genotyping of the selected SNPs was performed by real-time PCR using TaqMan probes. The significance of the differences in the distribution of genotypes was analyzed using Pearson’s chi-square test. Logistic regression modeling was performed to analyze the influence of the 164 investigated SNPs on ADDwoR. The *COL5A1* marker rs12722 turned out to be statistically significant (*p*-value = 0.0119), implying that there is a difference in the frequencies of TMJ ADDwoR. The distribution of rs12722 SNPs in the study group TT(66), CC(27), CT(31) vs. control group TT(45), CC(26), CT(51) indicates that patients with CT had an almost 2.4 times higher likelihood of ADDwoR (OR = 2.41) than those with reference TT (OR = 1), while rs13946 genotypes were shown to be insignificant, with a *p*-value of 0.1713. The *COL5A1* rs12722 polymorphism is a risk factor for ADDwoR in the Polish Caucasian population.

## 1. Introduction

The temporomandibular joint (TMJ) is a bilateral synovial articulation connecting the mandible to the temporal bones and allows complex movements in three dimensions. TMJ consists of the condylar head of the mandible, the glenoid fossa of the temporal bone, and the articular disc composed of densefibrousconnective tissue, surrounded by synovial fluid (Figure S1). The articular disc is a biconcave oval plate placed between the condyle and the mandibular fossa. It comprises the anterior and posterior band, with a thinner central part, called the intermediate zone. This anatomical structure is composed of fibrocartilage, while the extracellular matrix consists primarily of collagen (mainly type I and, to a lesser degree, type III), glycosaminoglycan, and proteoglycan [1]. In contrast to other synovial joints in the body, the articular surface of TMJ is covered not by hyaline, but fibrous cartilage. The unique feature of the latter is that it contains type I as well as type II collagen, while hyaline cartilage is made up of type II collagen [2,3]. It is important to mention that fibril formation of collagen types I and III are dependent on type V collagen. The latter is the regulatory fibrillar collagen, responsible for the optimal fibrillary formation and, as a consequence, proper tissue quality [4]. TMJ soft tissue structures are composed mainly of type I collagen (80%), but it is specifically collagen type V that plays a regulatory role in the process of fibrous tissue formation. TMJ disc divides the joint into two sections, each with its own synovial membrane. Other important structures are the temporomandibular, stylomandibular, and sphenomandibular ligaments. Similar to other joints, these ligaments play a protective role for TMJ structures (Figure S1).

They are composed of inextensible collagen fibers with a specific length. In the case of excessive forces affecting the joint, the ligaments can be irreversibly elongated, leading to functional impairment. Not only ligaments but, particularly, the articular disc and articular surfaces, when subjected to abnormal biomechanical forces, change their shape and function, which is referred to as arthropathy. Internal derangement (ID) is the arthropathy in which the articular disc has been displaced from the condylar head, and its origin is not yet completely understood. Furthermore, female adolescents are more prone to TMJ ID. Anterior disc displacement (ADD) with or without reduction (ADDwR or ADDwoR, respectively) is the most common type of internal derangement of TMJ [2,5,6]. In ADDwR, the articular disc glides out and into its proper, functional position during mouth closing and opening. By contrast, ADDwoR occurs when the disc slides anteriorly and slightly medially to a lower resting position, remaining trapped in the anterior joint recess Figure S2). The main causes of this condition are known as macrotrauma and microtrauma [5,6]. Macrotrauma refers to any sudden excessive force acting on TMJ that can damage joint structures directly or indirectly.

On the other hand, microtrauma results from small forces applied to the joint repeatedly over a long period of time. One example of repetitive microtrauma would be masticatory muscle hyperactivity in bruxism, which may occur during sleep (sleep bruxism) or day (awake bruxism) [7,8,9,10]. Moreover, correlation with anatomic variations and attachments of the lateral pterygoid muscle (LPM) and its influence in TMJ disc displacement were previously described [5].

In TMJ ADD, the anterior joint capsule of the lower joint space extends anteriorly together with the disc, considerably past the margin of the mandibular condyle [11]. This represents a significant alteration in the joint capsule [12].The displaced TMJ disc is supposed to revert at an earlier stage but, over time, may progress into a nonreducing form. Therefore, if the disc does not reduce, it then becomes displaced and may restrict the range of motion, causing TMJ dysfunction. The quality of a patient’s life experiencing ADDwoR deteriorates significantly, although there may be a necessity for medical intervention and intensive rehabilitation. There are reports of jaw asymmetry caused by unilateral ADDwoR in teenagers [5]. Furthermore, it has been proved that this type of mandibular asymmetry progresses over time and may require orthognathic surgery [5]. Osseous changes of the condylar head are significantly related to TMJ DD, and their severity rises with TMJ DD advancement. Further progression of ADDwoR may lead to severe bone resorption termed idiopathic condylar resorption (ICR), which is yet a poorly diagnosed disease with not yet well-understood underlying conditions. Nevertheless, the TMJ posterior band’s ligaments have been proved to play a significant role in preventing TMJ disc displacement [13].

Furthermore, it is logical to assume that the susceptibility to pathological changes of TMJ structures may be connective tissue constitution-dependent. The properties of connective tissue are conditioned by the quality of collagen fibers, determined not only by environmental, but also genetic factors. As previously mentioned, collagen type V plays a regulatory role in the process of fibrous tissue formation and is encoded by the *COL5A1* gene localized on the 9q34.3 chromosome.

There are reports describing the positive association between the single-nucleotide polymorphism (SNP) rs12722 or rs13946 in *COL5A1* and tendon or ligament injuries, such as, Achilles tendon pathology [14,15], anterior cruciate ligament (ACL) injuries [16,17], and tennis elbow [18]. Junkiert-Czarnecka et al. focused on the role of *COL5A1* in Ehlers–Danlos syndrome (EDS) in the Polish population [19]. EDS is a noninflammatory, heritable connective tissue disorder divided into 13 types according to the 2017 International Classification of Ehlers–Danlos syndrome. The classical type of Ehlers–Danlos (cEDS) syndrome is caused mainly by the defects in type V collagen, which is a quantitatively minor fibrillar collagen with wide tissue distribution. cEDS is characterized by joint hypermobility, skin hyperextensibility, and atrophic scars. In the study, nine new mutations of the *COL5A1* gene were found (eight missense mutations and one splice site), which, however, may be difficult to interpret and specify. Evaluation of these mutations by in silico tools indicates their pathogenicity. A similar study by Lin et al. investigated the co-occurrence of EDS and osteogenesis imperfecta (OI) in Chinese families [20]. Their results suggest that a combination of *COL5A1* and *COL1A1* mutations may lead to compound phenotypes of OI and EDS, while *COL1A1* (c.2010delT) may result in OI.

On the other hand, there have not been any studies investigating the association between previously mentioned *COL5A1* gene polymorphisms and TMJ internal derangements, including ADDwoR. It might be hypothesized that the gene-dependent susceptibility of TMJ to internal derangements is similar to other joints. In this study, we investigated the role of *COL5A1* rs12722 and rs13946 polymorphisms as potential genetic factors regulating the ADDwoR-mediated soft-tissue pathway. We focused on Caucasian patients, as the selected SNPs have not been investigated in European Caucasians for their role in TMD.

## 2. Materials and Methods

In this case–control study, the study group was recruited from patients who sought TMD treatment between 2014 and 2018 and presented to the Department of Dental Prosthetics, Pomeranian Medical University in Szczecin, Poland. It consisted of 124 Caucasian patients of both sexes. Each patient had an episode of ADDwoR no more than three months prior and signed an informed consent form before study registration. ADDwoR was diagnosed according to clinical examination, diagnostic criteria of the temporomandibular disorder questionnaire (DC/TMD), and CBCT/MRI [21]. The control group comprised 126 patients with no TMD problems according to DC/TMD. Additional exclusion criteria for both groups were as follows: pathological tooth mobility (grade 1 or more on the Hall scale), previous experience with occlusal splint therapy, not all areas of occlusal support present, coexisting pathology or inflammation within the jaws or head and neck muscles, concomitant metabolic diseases, or known connective tissue disorders.

SNPs selection: In this study, we chose to investigate the genetic role of the *COL5A1* gene rs12722 and rs13946 expression as a potential cause of the ADDwoR pathophysiological mechanism [22].

DNA isolation:Genomic DNA was isolated from oral epithelial cells using SWAB Genomic Extraction GPB Mini Kit (Genoplast Biochemical, Gdansk, Poland) according to the manufacturer’s instructions.

Molecular analyses:Genotyping of selected SNPs was performed by real-time PCR using TaqMan probes [23]. *COL5A1* rs12722 and rs13946 were analyzed using predesigned Applied Biosystems TaqMan real-time PCR assays (Applied Biosystems, Foster City, CA, USA).

The reaction mix for each sample consisted of GoTaq^®^ Probe qPCR Master Mix (Promega, Madison, WI, USA), TaqMan real-time PCR assays (Applied Biosystems, Foster City, CA, USA), and nuclease-free, deionized water, according to the manufacturer’s instructions.

The reaction mix, DNA, and no-template control (NTC) were pipetted into 384-well plates (Axygen Inc., New York, USA). Real-time PCR was performed on LightCycler 480 (Real-Time PCR System, Roche Diagnostics, Basel, Switzerland). Genotyping data were analyzed using LightCycler 480 Basic Software Version 1.5 (Roche Diagnostics, Basel, Switzerland).

Statistical analysis:Further on in the study of the polymorphisms, the odds were calculated in respect to the most frequent combination, with respective confidence intervals of 95%. A chi-squared at 0.05 confidence level test was performed in order to check for associations. The significance of the differences in the distribution of the genotypes was analyzed using Pearson’s chi-square test. Logistic regression modeling was performed to analyze the influence of the investigated SNPs on ADDwoR [24,25]. Data are presented as allele frequencies and odds ratio (OR) with a 95% confidence interval (CI). The Student’s *T*-test was performed to determine the age difference between the groups. *p* < 0.05 was considered to be statistically significant. The chi-square test was made for statistical analysis for sex distribution with a *p*-value of 0.129. Calculations were made using MATLAB version 8.6 (MathWorks, Natick, MA, USA, 2018) and RStudio software (RStudio Team (2020). RStudio: Integrated Development for R. RStudio, PBC, Boston, MA, USA, URL http://www.rstudio.com/, accessed on 7 July 2021).

The study was approved by the Ethics Committee of the Pomeranian Medical University in Szczecin, Poland, according to Good Clinical Practice (number KB-0012/88/14) and was conducted in accordance with the principles of the Declaration of Helsinki. The study was preceded by obtaining written formal consent from the patients who underwent dental examination and were enrolled in the examined group prior to oral swab collection.

## 3. Results

### 3.1. Patient Characteristics

The studied data were first analyzed using descriptive statistics in relation to the groups. There were no significant differences with regard to sex, but significant differences were found with regard to age distribution between the groups. Sex distribution was calculated with the χ^2^ test (*p*-value 0.129). The age of the control group was significantly higher in comparison to the case group (*p* < 0.001, *T*-test); no significant difference in sex distribution was found.

Complete demographic information and clinical parameters for the study populations are shown in Table 1.

### 3.2. Genotyping

**Figure 1:**
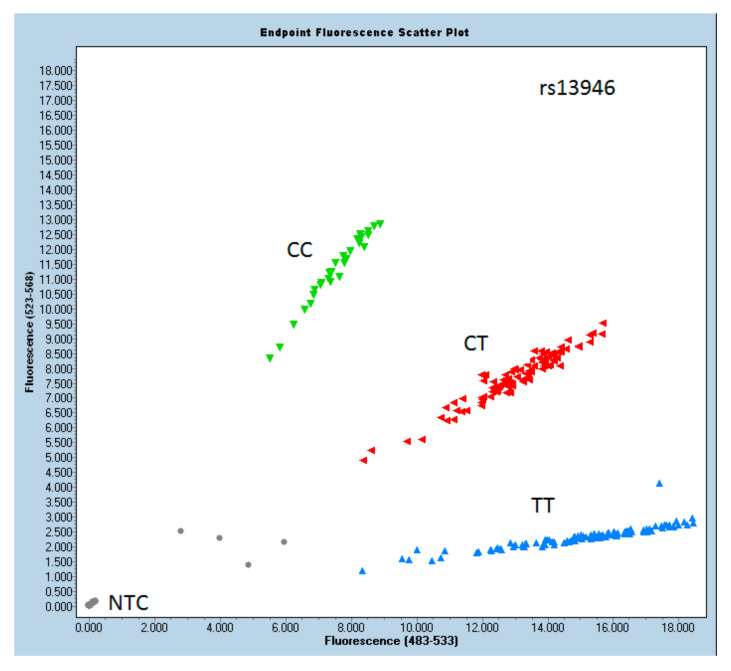
Allelic distribution plot of rs13946 genotypes in the study and control group.

**Figure 2:**
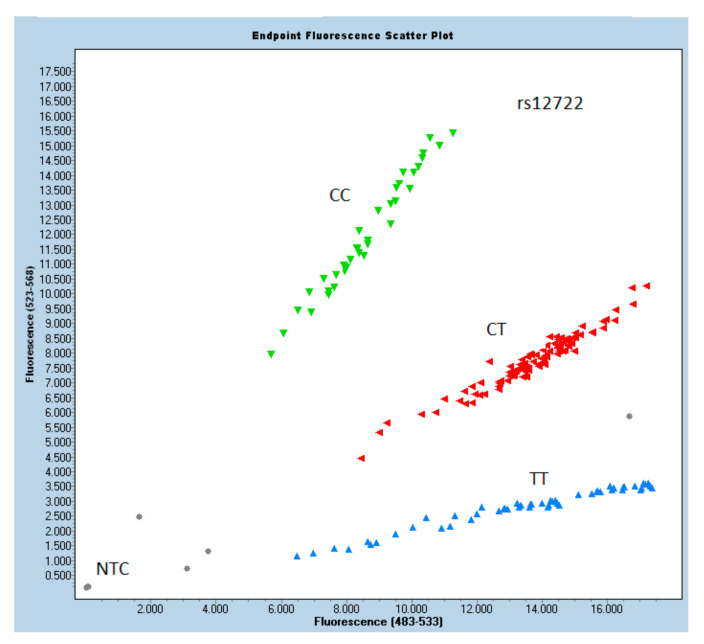
Allelic distribution plot of rs12722genotypes in the study and control group.

The results of the odds ratio analysis are shown in Table 2. In the study, the *COL5A1* marker rs12722 showed significant *p*-values (chi-squared), implying that there is a difference in the frequencies of temporomandibular joint disc dislocation. Patients with rs12722 genotype CT have an almost 2.4 times higher ADDwoR likelihood (OR = 2.41) than those with reference TT (OR = 1). On the other hand, the rs13946 genotypes do not seem to be related to disc dislocation, with insignificant *p*-values inthe allelic distribution plots of rs13946 and rs12722 genotypes, as presented in Figure 1 and Figure 2, respectively.

The multivariable logistic regression model showed a significant influence of CT allele on disc dislocation (Table 3). There was no significant effect of rs13946 in logistic modeling (data not shown).

## 4. Discussion

This is the first study of its type defining the relationship between *COL5A1* rs12722, rs13946, and ADDwoR. The main finding of our investigation wasa positive association between rs12722 and a higher risk of articular disc displacement.

The risk in patients with rs12722 genotype CT was almost 2.4 times higher (OR = 2.41) than the reference TT. This observation confirmed our hypothesis that TMJ, like other joints, might be prone to pathologies in the presence of particular genetic factors. On the other hand, a statistically nonsignificant *p*-value remained for rs13946. Furthermore, in the case of rs13946, patients with genotypes CC and CT (OR = 0.5668 and 0.641, respectively) seem less predisposed to ADDwoR than TT. Our observations are convergent with Pabalan et al.’s meta-analysis, where reduced risk effects on tendon–ligament injury among Caucasians were significant in rs12722 but not in rs13946 [26].

September et al. analyzed hereditary relationships in Australian and South African volunteers with Achilles tendinopathy [15]. Both groups were genotyped for *COL5A1* rs12722 and rs13946. They concluded that Achilles tendinopathy is associated with rs12722 but not rs13946 in Australian patients. This study also revealed that individuals with rs12722 CC genotype had a significantly lower risk of developing chronic Achilles tendinopathy than those with T allele (TT or TC) in Australian and South African patients. In contrast, our study revealed that patients with CC genotype had a higher risk of ADDwoR (OR = 1.4123) than TT (OR = 1), but lowerthan CT (OR = 2.41). This discrepancy may arise from different research objects (Achilles tendon), but also the different ethnic groups examined.

The study by Altinisik et al. enrolled 152 patients (107 female and 47 male) with lateral elbow tendinopathy (tennis elbow) and 195 healthy patients (152 female and 43 male), and demonstrated a high likelihood of tennis elbow in patients with specific alleles of the *COL5A1* gene, including both rs12722 and rs13946 SNP [18]. It was the first study (2015) to report rs12722 and rs13946 as a genetic risk factor for tennis elbow. These results are partly consistent with ours, where only the rs12722 polymorphism was associated with the joint dysfunction, while the female to male ratio was similar to our study. Mohmara et al. obtained different findings regarding *COL5A1* rs12722 and tennis elbow than those in Altinisik’s and our study [27]. There was no significant relationship between rs12722 and lateral elbow tendinopathy, while SNP *COL11A1* rs3753841 was associated with elbow tendon pathology. This discrepancy could stem from the fact that Mohmara’s study considered both medial and lateral tendons. The different diagnostic criteria may be another contributing factor.

Contrary to Altinisik’s study, relying on clinical criteria only, Mohmara included ultrasound imaging to confirm the diagnosis of elbow tendinopathy, increasing the objectivity of the study. Another study comparing 105 Chinese male patients suffering from an ACL injury with a control group of 110 healthy patients showed that *COL5A1* rs12722 is not significantly associated with anterior cruciate ligament injury [28]. Their observations indicated that *COL12A1* rs970547 and rs240736 are related to an ACL injury in the Chinese male population. This would suggest that rs12722 may be a risk factor for musculoskeletal soft tissue injuries. However, it has not yet been confirmed in different populations.

Interestingly, *COL12A1* rs970547 and rs240736 were not associated with ADDwoR in a study by Dalewski et al.; hence, the entire project was performed in Polish Caucasians [29]. A similar study by Stępień-Słodkowska et al. scrutinized the relationship between the rs12722 and rs13946 *COL5A1* polymorphisms and ACL ruptures in Polish recreational skiers [30]. The study group of 138 males with a diagnosed ACL rupture was compared to a control group represented by 183 injury-free male skiers. The results show a statistically significant under-representation of the CT haplotype in the study group compared to the controls. A higher frequency of *COL5A1*BstUI RFLP C/T (rs12722) and *COL5A1* DpnII RFLP C/T (rs13946) was observed in the control group. This finding indicates that these SNPs were, paradoxically, related to a minor risk of ACL injury in the group of healthy male skiers, while our study revealed that rs12722 CT genotype determined a higher risk of ADDwoR. Another study conducted by Lulińska-Kulik et al. reported that the C–C haplotypes rs12722–13946 turned out to be a protective factor for ACL injury in professional soccer players [31]. For comparison, our investigation showed that rs12722 CC genotypes were associated with a higher risk of ADDwoR. On the other hand, rs13946 CC genotype seems to play a protective role in disc displacement risk pathology.

Foster et al., in their in vivo study, reported that there is no relationship between the *COL5A1* rs12722 and tendon mechanical properties [32]. Dimensional, volumetric, and functional features of the patellar tendon in asymptomatic Caucasians were not confirmed. Nevertheless, tendon cross-sectional area measures were taken at 25%, 50%, and 75% of the tendon length and not 0% or 100%. Hence, there is a possibility that the volume underestimation possibly might have affected subsequent calculations of the elastic modulus.

The reason for the potentially conflicting evidence may arise from varying research methodologies comparing patients of different ethnic backgrounds. It may be assumed that research outcomes may differ depending on the joint type, race, ethnic group, gender, patient’s lifestyle, and body weight. One strength of our study is the significant *p*-value (0.0119) for rs12722, implying that there is a difference in the frequencies of TMJ articular disc dislocation. While the authors realize that the relatively small sample size represents a limitation in this study investigating genetics, the ADDwoR itself is a rather rare, or often underdiagnosed, condition. In further research, an increase in the number of patients, includingfrom different ethnic groups, should be considered to improve the statistical value of the correlation. The other limitation of our study is the unequal distribution of sex and age of the patients. This tendency is also present in other research analyzing TMD/disc displacement [33,34,35,36,37,38,39]. According to general statistics and results from similar studies [40,41,42,43,44,45,46,47,48], females are significantly more prone to TMD/disc displacement, and this consistency corresponds with our study. The reason for this fact is that sex hormones, primarily estrogen, seem to play an essential role in TMD pathophysiology [49,50,51]. Among the population consisting of 124 patients with ADDwoRwho sought TMD treatment in the years 2014–2018 in our department, males made up only 16.1%. Thus, the result in male populations may be unreliable. It would, therefore, be indicated to analyze mentioned polymorphisms on a larger scale in the future, possibly including more male patients.

Likewise, additional polymorphisms within *COL5A1–MIR608* rs4919510 may modulate the risk of Achilles tendon pathology [52,53,54]. Additionally, due to the limited data available ongenetic susceptibility to TMJ internal derangements, this might identify a new direction for further research in the future.

## 5. Conclusions

The major finding of this study is defining the association between *COL5A1* rs12722, rs13946, and ADDwoR. A positive correlation between rs12722 and a higher risk of articular disc displacement was confirmed in the population of Polish Caucasians. Patients with rs12722 genotype CT had an almost 2.4 times higher chance for ADDwoR (OR = 2.41) than those with reference TT (OR = 1). This observation confirmed our hypothesis that TMJ, like other joints, might be prone to specific pathologies in the presence of certain genetic factors. Moreover, the statistically nonsignificant *p*-value for rs12946 contributes new data to our body of knowledge, and also suggests a direction for further investigations.

## Figures and Tables

**Table 1:** Patient characteristics by the group.

		Total n = 250		Case n = 124		Control n = 126		*p*-Value Case vs. Control *
		N	%	N	%	N	%	
Sex	Females	200	80.00	104	83.87	96	76.19	0.129
	Males	50	20.00	20	16.13	30	23.81	
Age	Mean	38.15		32.36		43.86		<0.0001
	SD	17.03		13.03		18.56		

* Chi-square test.

**Table 2:** Odds ratio analysis.

		Case	Control	OR	CI 95%		*p*-Value **
rs13946—*COL5A1*							
reference	TT	50 (40.7%)	65 (52.4%)	1			
	CC	19 (15.4%)	14 (11.3%)	0.5668	0.2591	1.2397	0.1551
	CT	54 (43.9%)	45 (36.3%)	0.641	0.3737	1.1009	0.1070
rs12722—*COL5A1*							
reference	TT	66 (53.2%)	45 (36.9%)	1			
	CC	27 (21.8%)	26 (21.3%)	1.4123	0.7311	2.7284	0.1334
	CT	31 (25.0%)	51 (41.8%)	2.4129	1.3436	4.3333	0.0032 *

Genotypes: TT (thymine–thymine), CC (cytosine–cytosine), CT (cytosine–thymine); * significant; ** chi-square test.

**Table 3:** Logistic regression modeling using stepwise backward validation (model’s R2 Nagelkerke’s estimation = 0.075).

Rs12722	aOR	aOR 95% CI	*p*
CT	2.413	1.344–4.333	0.003
CC	1.708	0.849–3.439	0.133

## Data Availability

The data supporting the findings of this study are available from the corresponding author on request.

## Supplementary Materials

The following are available online at https://www.mdpi.com/article/10.3390/cells10092423/s1, Figure S1: Relation of the TMJ condyle and the disc in closed and open position, Figure S2: Anterior disc displacement without reduction—the sagittal view.

## Abbreviations

ACL	anterior cruciate ligament
ADD	anterior disc displacement
ADDwoR	anterior disc displacement without reduction
ADDwR	anterior disc displacement with reduction
CBCT	cone beam computed tomography
cEDS	classical type of Ehlers–Danlos syndrome
CI	confidence interval
CL	confidence level
CTS	carpal tunnel syndrome
DC/TMD	diagnostic criteria of temporomandibular disorder
EDC	estimated date of confinement
ICR	idiopathic condylar resorption
ID	internal derangement
LPM	lateral pterygoid muscle
MRI	magnetic resonance imaging
N	sample size
NTC	no template control
OI	osteogenesis imperfecta
OR	odds ratio
*p*-value	level of probability
PCR	polymerase chain reaction
SNP	single nucleotide polymorphism
TMD	temporomandibular disorder
TMJ	temporomandibular joint
