# Supplementary material for: COL5A1 RS12722 Is Associated with Temporomandibular Joint Anterior Disc Displacement without Reduction in Polish Caucasians
Source: Cells. 2021 Sep 14;10(9):2423. doi: 10.3390/cells10092423 (PMC8470511; doi:10.3390/cells10092423)
Supplement: Supplementary file 1 [file cells-10-02423-s001.zip › cells-1348142-supplementary.pdf]

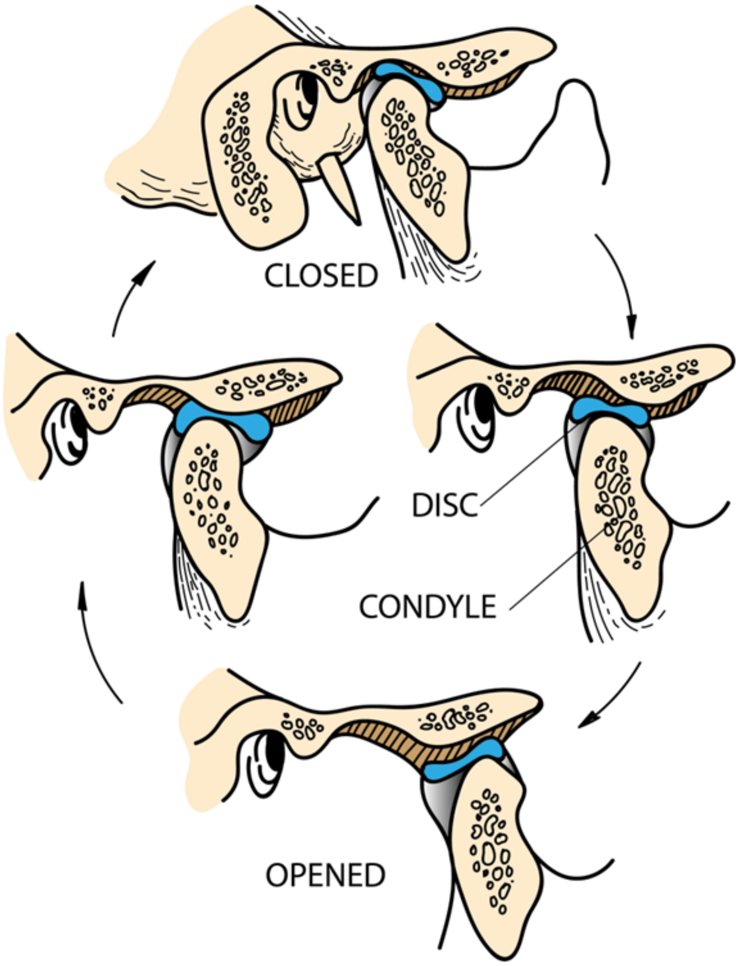

Figure S1.: Relation of the TMJ condyle and the disc in closed and open position.

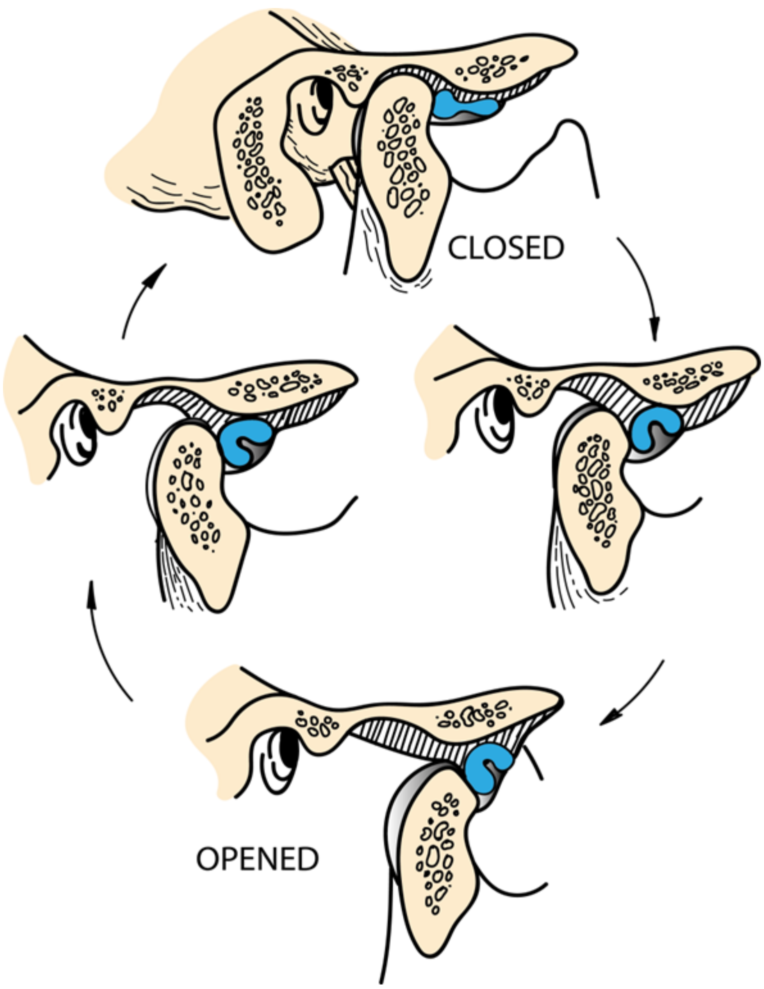

Figure S2. Anterior disc displacement without reduction - the sagittal view.
